# Direct, carryover, and maternal effects of ocean acidification on snow crab embryos and larvae

**DOI:** 10.1371/journal.pone.0276360

**Published:** 2023-10-18

**Authors:** William Christopher Long, Katherine M. Swiney, Robert J. Foy

**Affiliations:** Kodiak Laboratory, Alaska Fisheries Science Center, National Marine Fisheries Service, National Oceanic and Atmospheric Administration, Kodiak, AK, United States of America; University of Connecticut, UNITED STATES

## Abstract

Ocean acidification, a decrease in ocean pH with increasing anthropogenic CO_2_ concentrations, is expected to affect many marine animals. To examine the effects of decreased pH on snow crab (*Chionoecetes opilio*), a commercial species in Alaska, we reared ovigerous females in one of three treatments: Ambient pH (~8.1), pH 7.8, and pH 7.5, through two annual reproductive cycles. Morphometric changes during development and hatching success were measured for embryos both years and calcification was measured for the adult females at the end of the 2-year experiment. Embryos and larvae analyzed in year one were from oocytes developed, fertilized, and extruded *in situ*, whereas embryos and larvae in year two were from oocytes developed, fertilized, and extruded under acidified conditions in the laboratory. In both years, larvae were exposed to the same pH treatments in a fully crossed experimental design. Starvation-survival, morphology, condition, and calcium/magnesium content were assessed for larvae. Embryo morphology during development, hatching success, and fecundity were unaffected by pH during both years. Percent calcium in adult females’ carapaces did not differ among treatments at the end of the experiment. In the first year, starvation-survival of larvae reared at Ambient pH but hatched from embryos reared at reduced pH was lowered; however, the negative effect was eliminated when the larvae were reared at reduced pH. In the second year, there was no direct effect of either embryo or larval pH treatment, but larvae reared as embryos at reduced pH survived longer if reared at reduced pH. Treatment either did not affect other measured larval parameters, or effect sizes were small. The results from this two-year study suggest that snow crabs are well adapted to projected ocean pH levels within the next two centuries, although other life-history stages still need to be examined for sensitivity and potential interactive effects with increasing temperatures should be investigated.

## Introduction

The anthropogenic release of CO_2_ is causing an increase in atmospheric *pCO*_2_ which then dissolves into the oceans. The increased *pCO*_2_ in the oceans changes the carbonate chemistry, decreasing the pH and the saturation state of calcium content [1] in a process known as ocean acidification (OA). Since the beginning of the industrial revolution the pH of surface oceanic waters has dropped by approximately 0.1 units with a further ~0.3 unit reduction predicted by the end of this century [1–3]. High latitude waters are likely to acidify more rapidly than elsewhere, in part because CO_2_ is more soluble in colder waters and in part because of the upwelling of deep water rich in CO_2_ [4]. This change in ocean chemistry may have deleterious effects on many marine organisms, and, because of the accompanying decrease in calcium carbonate saturation state, calcifying marine organisms may be particularly vulnerable [5]. There is a great deal of variability both within and among marine taxa in their response to OA [6, 7] and this variability is projected to alter marine food webs and ecosystems [8].

Decapod crustaceans, many of which are valuable commercial species, are a calcifying marine taxa that can be strongly effected by OA [9]. When exposed to low pH many decapods respond through active ion transport in the gills, typically either H^+^/Na^+^ or Cl^-^/HCO3^-^ exchange, to maintain acid/base homoeostasis in their hemolymph [10], although this is not effective in all species or life history stages [11]. However, this active transport comes at an energetic cost which may divert energy away from other processes; this and other effects of OA may result in a range of effects including reduced hatching success [12, 13], slower growth [14–16], higher mortality [17–19], deformities [20, 21], immune disruption or increased disease rate [22, 23], reduced calcium content or hardness in the exoskeleton [18, 24, 25], and altered behavior [26, 27] in many species. However, other species are highly tolerant of OA [28, 29]. For species that are negatively affected, reductions in ocean pH are predicted to decrease population size and any fisheries that depend on them [30–34].

Not only does OA have direct effects on marine species, but carryover effects, where exposure at one life history stage affects fitness in a subsequent life history stage, frequently occur. For example, for both red king crab, *Paralithodes camtschaticus*, and the spider crab *Hyas araneus* exposure of embryos to high *pCO*_2_ reduces survival at the larval stage [35, 36]. Maternal effects, a sub-type of transgenerational effects, are when exposure of the mother during oocyte development affects larval or juvenile performance and are a particular sub-set of carryover effects [37]. Sometimes, as in the case of the mussel *Musculista senhousia*, maternal effects can be positive [38] indicating adaptive potential, but in other species maternal effects can be negative [13] suggesting reduced maternal investment in oocytes under stressful conditions. Therefore, it is necessary to quantify if and to what degree carryover effects occur for a particular species to avoid over or underestimating the effects of OA.

Snow crabs, *Chionoecetes opilio*, are a high latitude species with populations ranging across the North Pacific from Japan to the United States and up into the Arctic in the Beaufort and Chukchi Seas, another in the Gulf of St. Lawrence, and another in the Barents Sea, where it is an invasive species [39, 40]. It is also an important fisheries species; in 2018 the ex-vessel value was $75.2 million in the Bering Sea fishery alone [41]; however, the population in Bering Sea recently crashed [42], most likely caused by record warm waters [43]. Across its range, even where it is not fished, snow crab can be a dominant biomass in the benthic community and play an important role as a primary/secondary benthic predator within the ecosystem [44]. Snow crab have a life history that is typical of many decapod crustaceans. Pubescent females undergo a terminal molt to maturity and mate before extruding a clutch of eggs in the early spring [45]. Eggs are brooded for either approximately a year or two years (depending on temperature) and hatch in the late spring [46–50]. The larvae are planktivorous and pass through two zoeal and one megalopea stage before settling to the benthos and molting to the first crab stage [51]. Although no work has been done to date examining the effects of OA on snow crab, a congener species, the southern Tanner crab, *C*. *bairdi*, is highly vulnerable to OA; OA can reduce hatching success by over 70% [13], affects larval fitness, and decreases juvenile growth and survival [52], although there are no direct effects on larval size and starvation-survival or calcification [53]. Finally, in mature female Tanner crab, OA increases hemocyte mortality and decreases intracellular pH [22] and causes internal and external erosion of the exoskeleton which substantially weakens it [54].

Carbonate chemistry in the eastern Bering Sea varies spatially and seasonally; however, a complete picture of the system is lacking due to prolonged ice cover for much of the year. After ice retreat in the spring the system becomes stratified and respiration in the bottom waters, which is the habitat for ovigerous females and their embryos, increases the *pCO*_2_ from approximately 400ppm in the late spring to 1400–1600 ppm in the early summer [55]. In general, on the shelf, calcium carbonate saturation states are highest in shallower waters and increase with depth and also grade from high to low along a south to north [56]. Current models predict average shelf pH level below 7.8 for about half the year; by the year 2100 the average pH is predicted to be below 7.5 for more than 40% of the year [57]. Larvae are planktonic and are distributed mostly in the mixed layer above the thermocline [51, 58] from late spring through early fall. Photosynthesis during the spring bloom can reduce the *pCO*_2_ down to 150ppm by May which then slowly increases back up to atmospheric partial pressures by the early autumn [56].

Given its high value as a commercial species, its importance to benthic ecosystems, and the vulnerability of closely related species, it is an important target species for OA research. In this paper, we present the results of a study to determine both the direct and maternal effects of OA on snow crab embryos, fecundity, as well as the direct and carryover effects on larvae.

## Methods

### Overview

**Fig 1:**
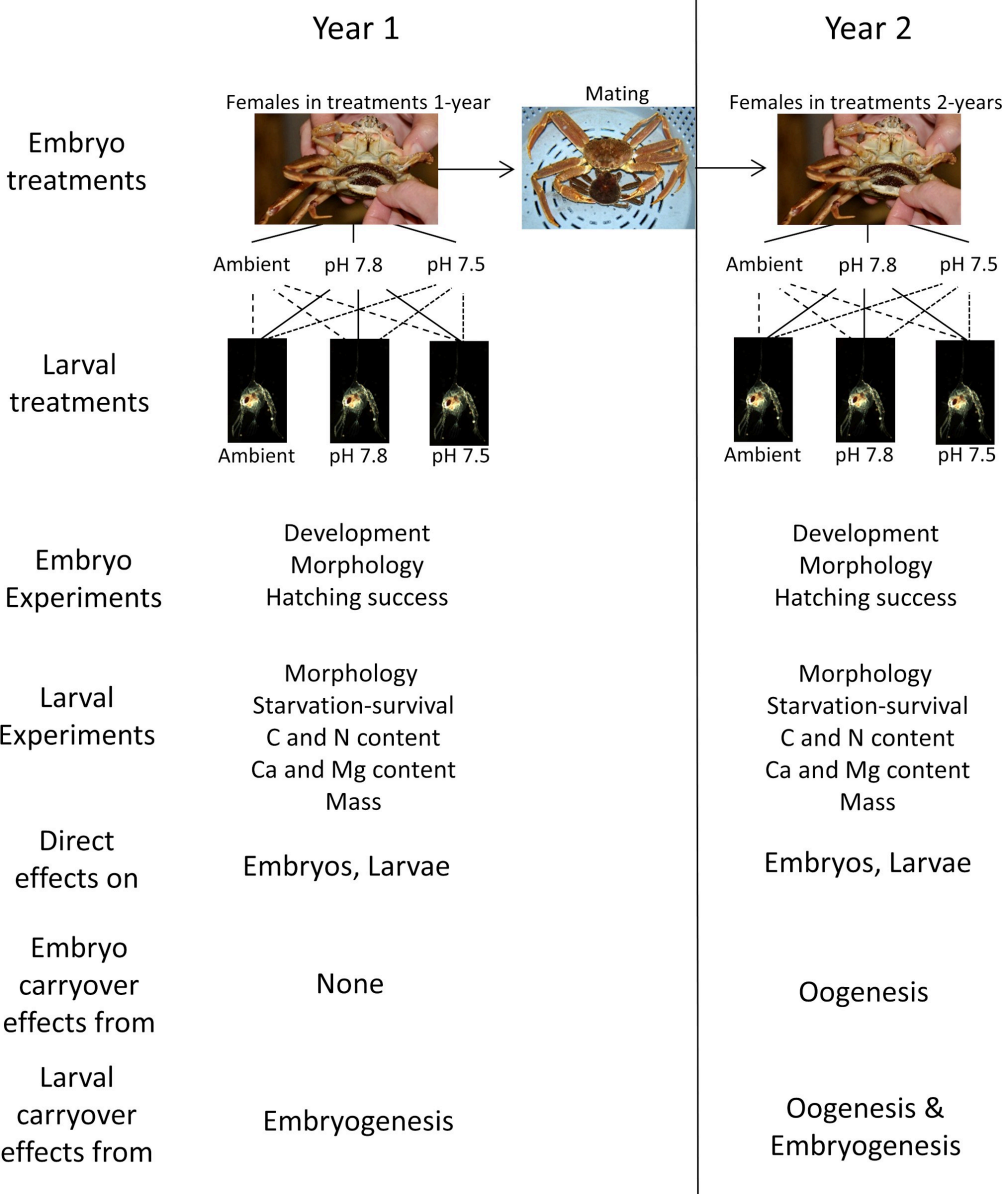
Experimental design. Schematic of design of experiment to determine the effects of ocean acidification on snow crab embryos and larvae. Arrow indicates that the same females were held over the full 2-year experiment and were mated between years. Larval treatments were fully crossed (9 total combinations) but only one treatment expansion is shown for sake of space.

Female crabs in this experiment were held through two brooding/hatching cycles (Fig 1). In the first year, wild-extruded embryos were exposed to three pH treatments to quantify the direct effects of OA on embryo development. At hatching, larvae were collected individually from each female and hatching success and fecundity were determined. After hatching, females were allowed to mate with a male, extruded a new clutch of eggs, and were held in the same treatment pH as the first year throughout embryo development and larval hatching. The second year allowed us to examine the combination of maternal effects (i.e. carryover effects from oogenesis) and direct effects on embryo development and hatching success. In both years we also performed a series of experiments on the larvae in a design that fully crossed embryo pH treatment with larval pH treatment. The first year’s experiments allowed us to determine the direct effects of OA on the larvae and the carryover effects from embryogenesis whereas the second year’s allowed us to determine both the direct effects of OA on the larvae and the carryover effects from both oogenesis and embryogenesis combined (Fig 1).

### Female collection and holding

Female snow crab with newly-extruded uneyed eggs and mature male snow crabs were captured on the 2014 eastern Bering Sea trawl survey [59] and transported to the Kodiak Fisheries Research Center in coolers packed with damp burlap and ice packs via airplane from Dutch Harbor on July 16, 2014. Females were held in tanks with flow-through sea water at ambient pH and salinity chilled to 2°C briefly before the beginning of the experiment on August 6, 2014; males were held in the same conditions (ambient pH) until needed for mating (see below). Sand filtered seawater at ambient salinity from Trident Basin intakes at 15 and 26m was used throughout this experiment. Throughout holding and the entire experimental period crabs were fed a diet of frozen herring and squid to excess twice a week. Twenty-five crabs were randomly assigned to each of one of three pH treatments based on projected global future ocean pH levels: current surface Ambient (~pH 8.1), pH 7.8 (projected for 2100), and pH 7.5 (projected for 2200). Throughout this manuscript, “Ambient” (capitalized) will be used to refer to the treatment in these experiments to differentiate it from other uses, When this experiment was planned there were no regional carbonate system projections available; however, these levels are currently relevant in Bering Sea with seasonal lows of pH around 7.5 [55]. We also wanted to be able to directly compare results with our previous experiments on the closely related Tanner crab [13, 53] and so used the same pH treatments. Only healthy crabs with full clutches and no more than 2 missing limbs were used. During year 1, 15 randomly selected crabs from each treatment were sampled for embryo development and hatching (see below). The number of crabs that survived the first year out of the 15 starting were 12, 10, and 9 in the Ambient, pH 7.8, and pH 7.5 treatments respectively. After the first year only 3 crabs, 1 in the Ambient treatment and 2 in the pH 7.8 treatment, did not extrude new clutches of eggs. During year two, 14 females, comprised of the crabs from the original 15 that had survived the first year and extruded a new clutch of eggs, plus additional crabs from the crabs not sampled the previous year were sampled for embryo development and hatching in each treatment. Females that were not selected for sampling were held in the same manner as those which were.

During the majority of the experiment, female crabs within each treatment group were held communally in experimental holding tanks, one per treatment, (0.6 m x 1.2m x 0.6m) supplied with flow-through water at the appropriate pH (see below for details on water acidification). A temperature of 2°C was maintained in each tank with a recirculating chiller. Larval experimental tanks also received water from this system and they were kept at 3 ± 0.5°C by recirculating chillers. Both years, as crabs neared the time for hatching they were transferred to individual 68 L tubs. This was done so that hatching data could be collected for each individual female. All hatching tubs received recirculating water from a 2000 L head tank (one per treatment) that received flow-through seawater, was adjusted to the appropriate pH (see below), and maintained at 2°C with a recirculating chiller. Although this design does not allow us to determine if there was a tank effect as crab were held either together or received water from a communal head tank, the fact that the embryos are held under the abdominal flap and protected by each female is sufficient isolation to treat each as an independent replicate. In the first year, after a female stripped her pleopods (cleaned off the empty egg cases and remaining unhatched eggs in preparation for extruding a new clutch), she was held with a male as a potential mate to ensure there was no potential for sperm limitation in the second year; however, as female snow crab can store sperm we were unable to determine if a female was mated and whether fresh sperm from that mating, stored sperm, or a combination of stored and fresh sperm was used to fertilize her new clutch. Males were randomly assigned to females and no male was used as a potential mate for more than two females. After all the females had extruded a new clutch of eggs, they were transferred back to communal holding in the experimental holding tanks.

### Water acidification and chemistry

In both the experimental tanks and the hatching tubs the seawater was acidified using CO_2_. The water flowing into the holding tanks was acidified as described in [52]. In brief, water was acidified down to ~pH 5.5 by bubbling CO_2_. This water was mixed with seawater in head tanks using peristaltic pumps controlled with Honeywell controllers and Durafet III pH probes in the head tanks to achieve the nominal pH. Water from the head tanks then flowed into the experimental holding tanks. When crabs were transferred to the hatching tubs, the head tank supplying the water was acidified by bubbling CO_2_, the flow of which was controlled by Honeywell controllers and Durafet III pH probe in the head tanks. Temperature and pH (free scale) were measured daily in experimental units for both the females and the larvae using a Durafet III pH probe calibrated with TRIS buffer [60]. Water from the head tanks was sampled once per week and samples were poisoned with mercuric chloride and analyzed for dissolved inorganic carbon (DIC) and total alkalinity (TA) at an analytical laboratory. DIC and TA were determined using a VINDTA 3C (Marianda, Kiel, Germany) coupled with a 5012 Coulometer (UIC Inc.) according to the procedure in [61] using Certified Reference Material from the Dickson Laboratory (Scripps Institute, San Diego, CA, USA; [62]). The other components of the carbonate system were calculated in R (V3.6.1, Vienna, Austria) using the seacarb package [63].

Treatment pHs for the two acidified treatments exactly averaged the target levels across two years of treatment and water temperatures were well maintained throughout the experiment (Table 1). The Ambient pH treatment was supersaturated with regards to both calcite and aragonite while the pH 7.8 treatment was undersaturated with regards to aragonite and the pH 7.5 treatment was undersaturated with regards to both (Table 1).

**Table 1:** Water physical and chemical parameters in female experimental tanks.

Treatment	Temperature	pH_F_	*pCO*_2_	HCO_3_^-^	CO_3_^-2^	DIC	Alkalinity	Ω_Aragonite_	Ω_Calcite_
			μatm	mmol/kg	mmol/kg	mmol/kg	mmol/kg		
Ambient	2.09 ± 0.32	8.11 ± 0.08	362.18 ± 68.33	1.90 ± 0.05	0.09 ± 0.02	2.01 ± 0.04	2.11 ± 0.02	1.36 ± 0.23	2.19 ± 0.37
pH 7.8	1.97 ± 0.30	7.80 ± 0.02	760.98 ± 43.95	2.00 ± 0.04	0.05 ± 0.00	2.09 ± 0.05	2.09 ± 0.21	0.69 ± 0.04	1.11 ± 0.06
pH 7.5	2.05 ± 0.31	7.50 ± 0.02	1548.29 ± 102.11	2.04 ± 0.06	0.02 ± 0.00	2.15 ± 0.06	2.11 ± 0.02	0.36 ± 0.02	0.57 ± 0.04

Water parameters in experimental tanks during snow crab experiments. Temperature and pH were measured daily (N = 681 per treatment), dissolved inorganic carbon (DIC) and alkalinity were measured weekly (N = 98 per treatment), and all other parameters were calculated. Values are mean ± 1 SD.

### Embryo development

Embryo development was monitored throughout both years. Once a month, approximately 20 eggs were randomly sampled from each female. The embryonic developmental stage was determined per Moriyasu and Lanteigne [47]. In, brief, uneyed eggs were stained for 5 minutes with Bouin’s solution to facilitate observation of the external morphology of the embryos; eyed eggs were not stained. The embryonic stages were determined under a stereo microscope at ~63x magnification. Additionally, digital images of ten fresh eggs from each female were taken with a digital camera attached to the same microscope at a total magnification of 63x. Measurements were made as per Swiney et al. [13]. In brief, using image analysis software (Image Pro Plus Version 7.0.1.658, Media Cybernetics, Inc., Rockville, Maryland USA), egg area and diameters (maximum, minimum, and average) were measured. Once embryos were discernable, embryo areas and yolk areas and diameters (maximum, minimum, and average) were also measured. Lastly, when embryos become eyed, eyespot area and diameters (maximum, minimum, and average) were measured. Percent area yolk (PAY) was calculated as: $PAY=100\frac{yolk\;area}{egg\;area}$. For each female, within each sampling period, the 10 measured embryos were averaged prior to analysis. In May 2015, all but five of the females had already extruded new clutches of eggs; as there were so few females, measurements made in May on their embryos were not included in either the analysis of embryo stage or embryo morphometrics.

Embryo stage was analyzed separately for each year using an ANOVA with pH treatment fully crossed with month and female (nested within treatment) as factors. In this and in all ANOVA-type analyses the assumptions normality and homogeneity of variance were checked with Anderson-Darling and Levene’s tests respectively. Because ANOVA analyses are highly robust to deviations from the assumption of normally [64], we interpret the ANOVA results directly in cases where the data failed to meet that assumption. In cases where the data was heteroscedastic, we rejected the ANOVA at an α value lower than that from the test of homogeneity of variance [65]. Embryo morphometrics (averaged for each female within each sampling date) were analyzed separately for each year and normalized (expressed in terms of their z-score) prior to analysis. Morphometrics were analyzed using permutational MANOVA (PERMANOVA) on a Euclidian distance resemblance matrix with pH treatment fully crossed with month and female (nested within treatment) as factors. The assumption of homogeneity of dispersion was tested with a permutational analyses of multivariate dispersions (PERMDISP). Data were visualized using a non-metric multidimensional scaling plot (MDS).

### Fecundity, hatching success, and mineral content

Before hatching began, crabs were moved into individual tubs and larvae were collected in a net at the outflow of each tub. Each day, every net was checked and the number of larvae estimated. If there were fewer than ~100 larvae the larvae were counted; if there were more than 100, the number hatched was estimating using dry mass. The larvae were collected, rinsed briefly in DI water, dried to a constant mass at 60°C and weighed. For each female, five times throughout hatching (approximately every other day) a subset of 50 larvae were counted out, dried to a constant mass, and the dry mass was determined. The average larval mass for each female was calculated and used to estimate the total number hatched each day. On a number of occasions, larvae were collected live for experiments (see below) and could not be dried. In these cases, the number of larvae was estimated by taking 3–4 subsamples of known volume, counting the number of larvae in each, and using the average concentration of larvae to calculate the total number.

After hatching was complete and females had stripped their pleopods, the debris was collected and examined microscopically and the total number of unhatched eggs, and viable and non-viable larvae were estimated from volumetric subsamples. The percent non-viable larvae and unhatched eggs were also calculated for each female. Fecundity was defined as the number of viable larvae hatched. Hatching success was defined as the percent of the total estimated number of embryos (viable larvae + non-viable larvae + unhatched eggs) that hatched into viable larvae. Non-viable larvae were defined as larvae which hatched but failed to molt from the pre-zoeal to the first zoeal stage. At the end of the final year all females were sacrificed and the calcium and magnesium content in their exoskeletons was determined in an analytical laboratory from an approximately 2 x 2 cm sample of the carapace taken from the posterior margin.

Fecundity, hatching success, percent non-viable larvae, percent unhatched eggs, and carapace calcium and magnesium contents were all analyzed with a one-way ANOVA with pH treatment as the factor.

### Larval experiments

Larval experiments were identical to those in Long et al. [53], except that the water temperature was kept lower during these experiments because snow crab have a lower temperature range than Tanner crab (*Chionoecetes baridi*). Larval experiments were conducted in inserts made from cut pieces of PVC piping with mesh bottoms placed inside larger treatment tanks. Each insert received water flow of 100 ml/min via a recirculating submersible pump. Survival experiments were performed in inserts with a volume of ~1 L and all other experiments in inserts with a volume of ~2 L. Larvae were unfed during all experiments. During hatching females were held in individual containers and the number of larvae hatched each day was estimated. Larvae experiments were started once 3 females within a treatment were at or near peak hatch. Newly hatched larvae for use in the experiments were collected overnight and pooled from 3 females per treatment per year; because females from the different treatments were not all synchronous in their hatching each year, there was some small variation, 7 days in 2015 and 14 days in 2016, among embryo treatments in the start date for the different experiments. For each experiment, embryo treatment was fully crossed with larval treatment for a total of 9 treatments. Five replicates (inserts) were run within each of those 9 treatments for a total sample size of 45 for each experiment.

For starvation survival experiments, 20 larvae were counted out and placed in inserts. They were monitored daily and dead larvae were removed. Each trial was ended when all larvae had died or after 7 weeks had passed (no trial had more than 1 surviving larvae in it when the experiment ended). Mortality data were fit to a series of models using maximum likelihood and assuming a binomial distribution of data. In all models, mortality was modeled with a power formulation of the logistic regression:

$$P_{m}=\frac{1}{1+(\frac{t}{t_{50}}{)}^{s}}$$

Where *P*_*m*_ is the probability of mortality at time *t*, *t*_*50*_ is the time to 50% mortality (also known as the LT_50_) and *s* is a slope parameter. A number of models were fit to the data in which *t*_*50*_ and s were allowed to vary linearly with embryo treatment, larval treatment and their interaction. The Akieke’s Information Criterion corrected for small sample size (AIC_c_) was calculated for each model and the most parsimonious model selected. Models whose AIC_c_s differed by less than two were considered to have equal weight [66]. Data from each year was analyzed separately.

Condition experiments and calcification experiments were performed in separate trials but using similar methods. When beginning each experiment, samples from the initial pooled larvae were taken and analyzed for dry mass, carbon and nitrogen (CN) content, and calcium and magnesium content as described below. For both experiments, ~300 larvae (estimated volumetrically) were transferred into each insert. Larvae were checked daily and dead larvae were removed by siphoning them off the bottom. The larvae were exposed to treatment water for 7 days. From the condition experiment, 50 larvae per replicate were sieved, rinsed briefly in fresh water, and dried. The mass was determined and the average larvae dry mass calculated. The remaining larvae in each insert were sieved, rinsed, dried, and recombined with the 50 larval used to determine the dry mass. All the dried larvae from each insert were ground together and the CN contents were determined on a subsample (one sample per insert). The carbon and nitrogen content was determined at the University of Santa Barbara using the Dumas combustion method and an automated organic elemental analyzer [67]. For the calcification experiment, the larvae from each replicate were sieved, rinsed, and dried and analyzed for calcium and magnesium content at Gel Laboratories via inductively coupled plasm-atomic emission spectrometry. Larval dry mass, carbon, nitrogen, calcium, and magnesium contents, and the C:N ratios were analyzed with fully crossed 2-way ANOVAs with Embryo and Larval treatments as the factors for each year. Where differences were significant Tukey’s post hoc test was used to determine differences among treatments.

Larval morphometry was determined by measuring 15 larvae from the larvae pooled for the condition experiment from each treatment from each year. Digital micrographs were taken of each larvae using a stereo microscope and six measurements were made on each larvae using Image-Pro Plus v. 6.00.260 (Media Cybernetics, Inc., Bethesda, MD, USA): carapace width (including spines), lateral spine length, dorsal spine length, rostro-dorsal length, rostral spine length, and protopodite length as per Long et al. [53]. Measurements were normalized (expressed in terms of the z-scores) before analysis. Morphometry was analyzed using an analysis of similarity (ANOSIM) and visualized using a non-metric multidimensional scaling both performed on a similarity matrix using Euclidean distance. Where there were significant differences among treatments a Similarity Percentage (SIMPER) analysis was performed on the raw (non-normalized) measurements to determine which measurements most strongly influenced the differences among treatments.

## Results

### Embryo development

**Fig 2:**
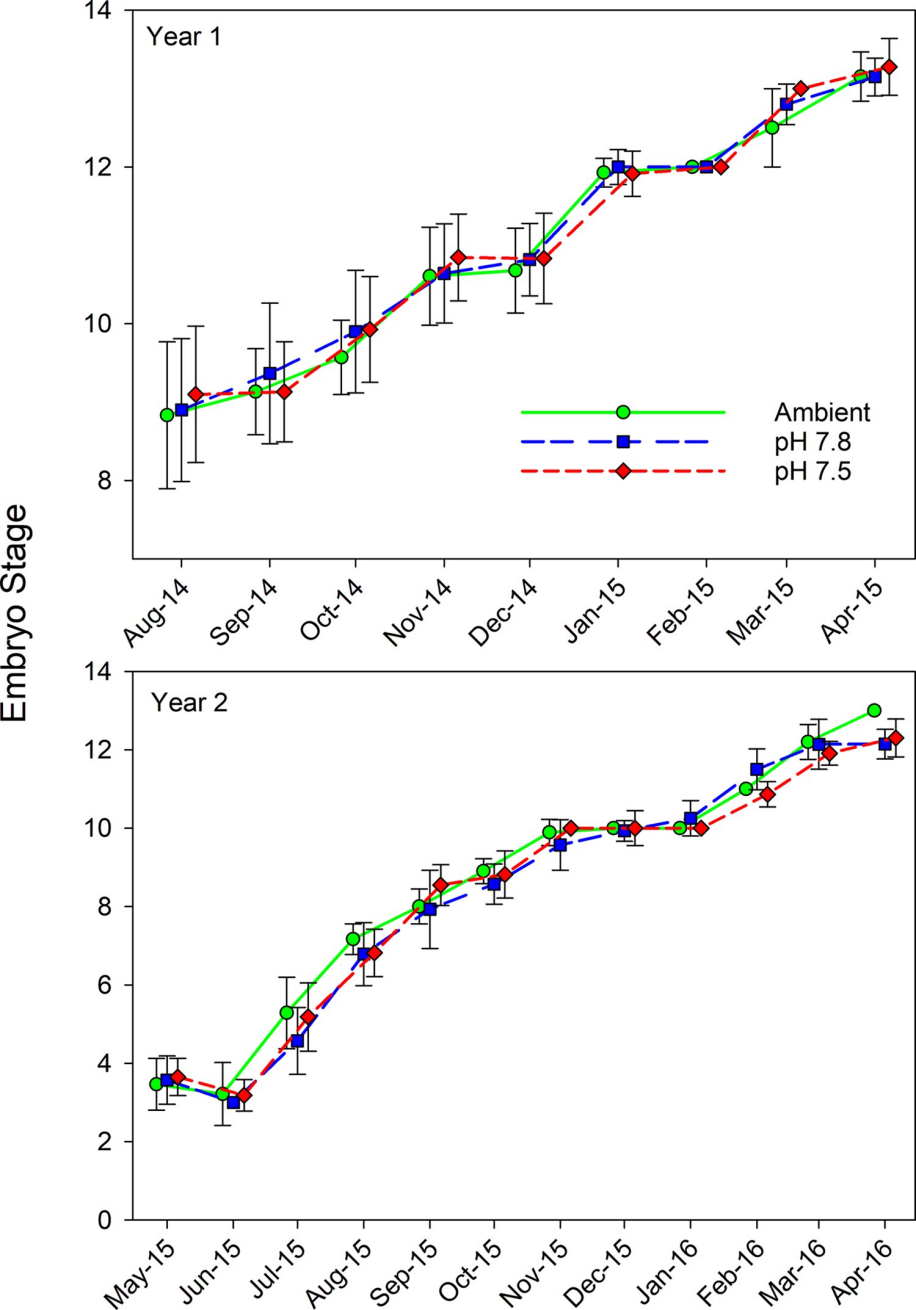
Effect of pH on snow crab embryo stage. Effects of three different pH treatments on the stage of embryo development in snow crabs over two successive brooding cycles (Year 1 and Year 2). Symbols are the mean stage for each month and error bars are one standard deviation. All embryos were sampled on the same day; however, treatments are offset for clarities sake.

The average embryo stage increased over time in both years of the study but did not differ among pH treatment (Fig 2). In the second year, stage data violated the assumptions of normality and homogeneity of variance, likely because in a few months all the females within one or more treatments were at the same stage, and so the α value for ANOVA interpretation was set to the p-value from Levene’s test (p = 1×10^−15^). Stage differed among months in both years (Year 1: F_8,227_ = 2.412, p < 0.0005; Year 2: F_11,326_ = 1,141.6, p > 5×10^−324^) but not among treatments (Year 1: F_2,227_ = 2.412, p = 0.092; Year 2: F_2,326_ = 1.097, p = 0.335). The interaction between month and treatment was not significant in either year (Year 1: F_16,227_ = 0.535, p = 0.928; Year 2: F_22,326_ = 2.838, p = 3.3×10^−5^).

**Fig 3:**
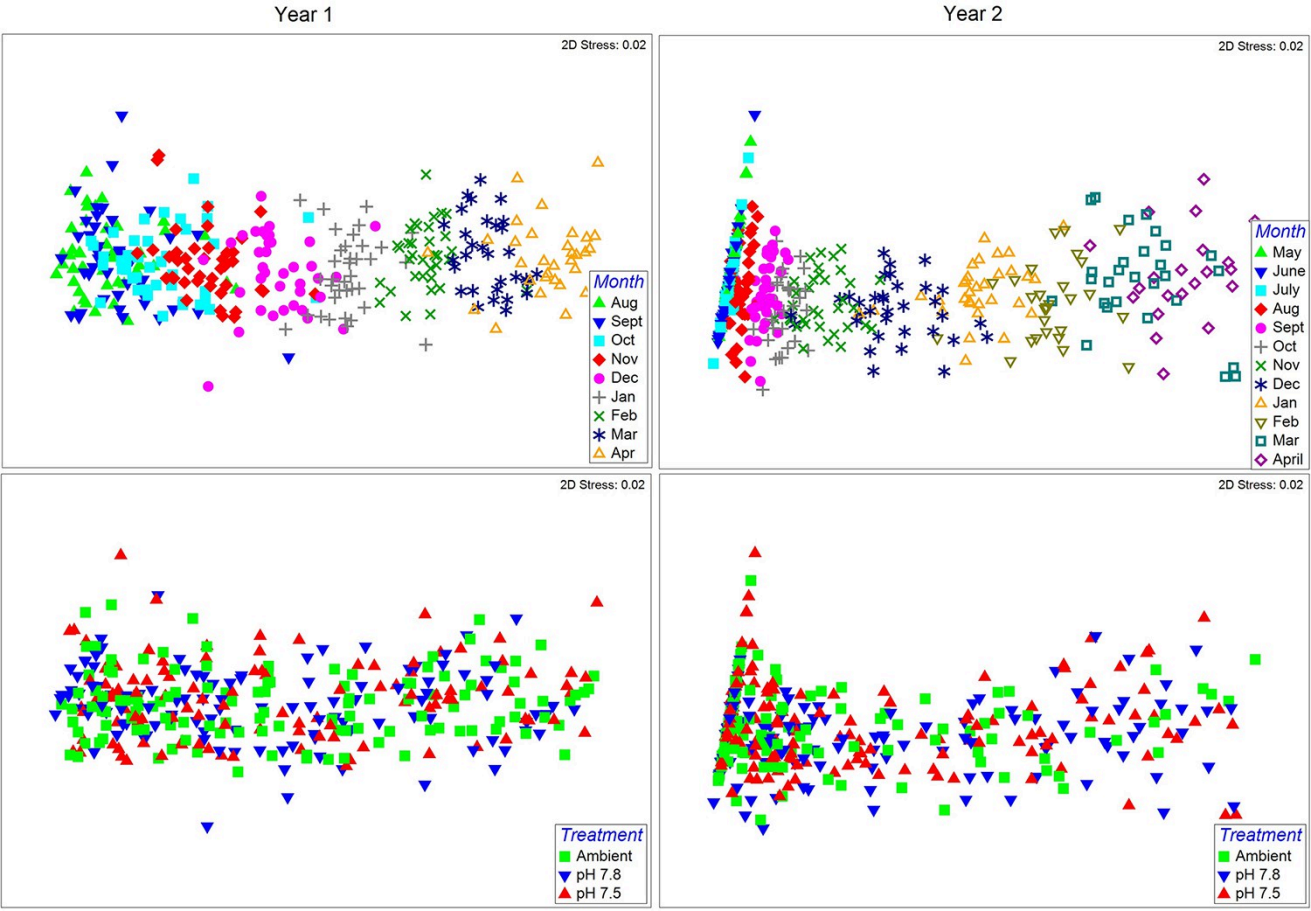
Effect of pH on snow crab embryo morphometrics. Non-metric multidimensional scaling plots of snow crab embryo morphometrics measured monthly during two brooding cycles (years) and reared in 3 different pH treatments. Plots on the left represent the year 1 embryos (first brooding cycle), and those on the right represent year 2 embryos. Top row of plots graph the data by month to show embryo development and the bottom 2 rows graph the data by pH treatment.

Embryo morphometrics showed a clear progression in development among months (Fig 3) in both year 1 (PERMANOVA; Pseudo-F_8,272_ = 467.63, p = 0.001) and year 2 (Pseudo-F_11,387_ = 549.29, p = 0.001). In the second year only, dispersion also differed among the months (PERMDISP, F_11,387_ = 4.469, p = 0.001). A significant PERMANOVA and PERMDISP indicates that either location and dispersion differ among months, or just dispersion does; examination of the nMDS in this case shows that the former is almost certainly the case [68]. Similar to the first year, there is an expected developmental progression with completely non-overlapping months. However, dispersion at the earliest embryo stages when the embryo and eyes are not yet measurable are much smaller than later stages when they are. There was no difference among treatments in either year 1 (Pseudo-F_2,272_ = 1.065, p = 0.385) or year 2 (Pseudo-F_2,327_ = 549.29, p = 0.469), but there was a significant interactive effect in both years (year 1: Pseudo-F_16,272_ = 1.451, p = 0.001; year 2: Pseudo-F_22,327_ = 1.6146, p = 0.003); however, there was no clear pattern discernable. In the first year, a post-hoc PERMANOVA indicated a significant difference between pH 7.8 and Ambient embryos in October and a difference between pH 7.8 and the other two treatments in April; in the second year there was a significant difference between Ambient and pH 7.5 embryos in December but no other month. Given large number of contrasts (60 total), the lack of any pattern either within or between years, and the strong overlap of the treatments in the MDS plots in both years, we interpret these differences as representing type I statistical errors.

### Fecundity, hatching success, and mineral content

**Fig 4:**
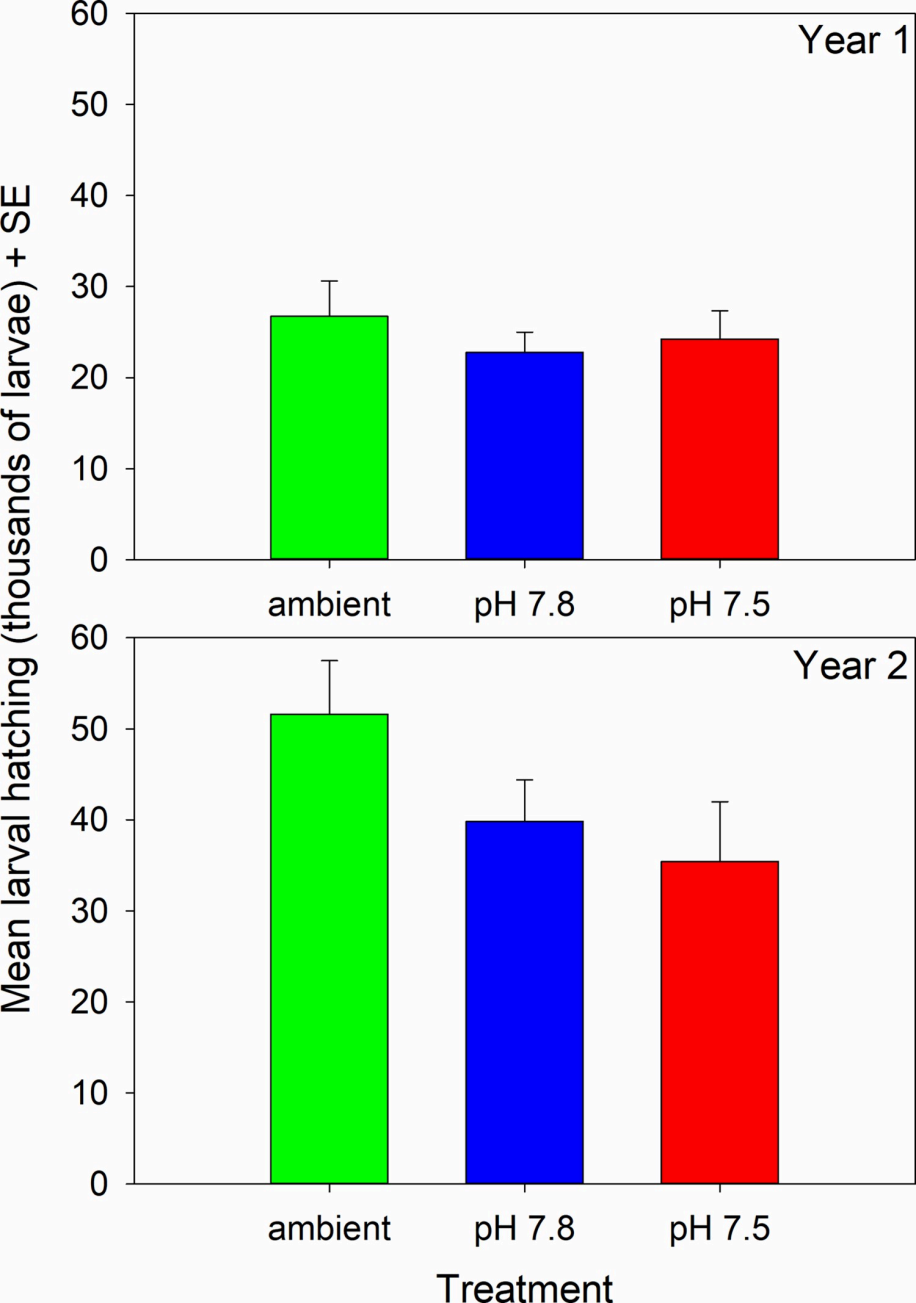
Effect of pH on snow crab fecundity. Fecundity, defined as the number of viable larvae hatched, in females held in 3 different pH treatments over 2 years. There are no statistically significant differences among treatments in either year. Error bars are plus 1 standard error.

**Fig 5:**
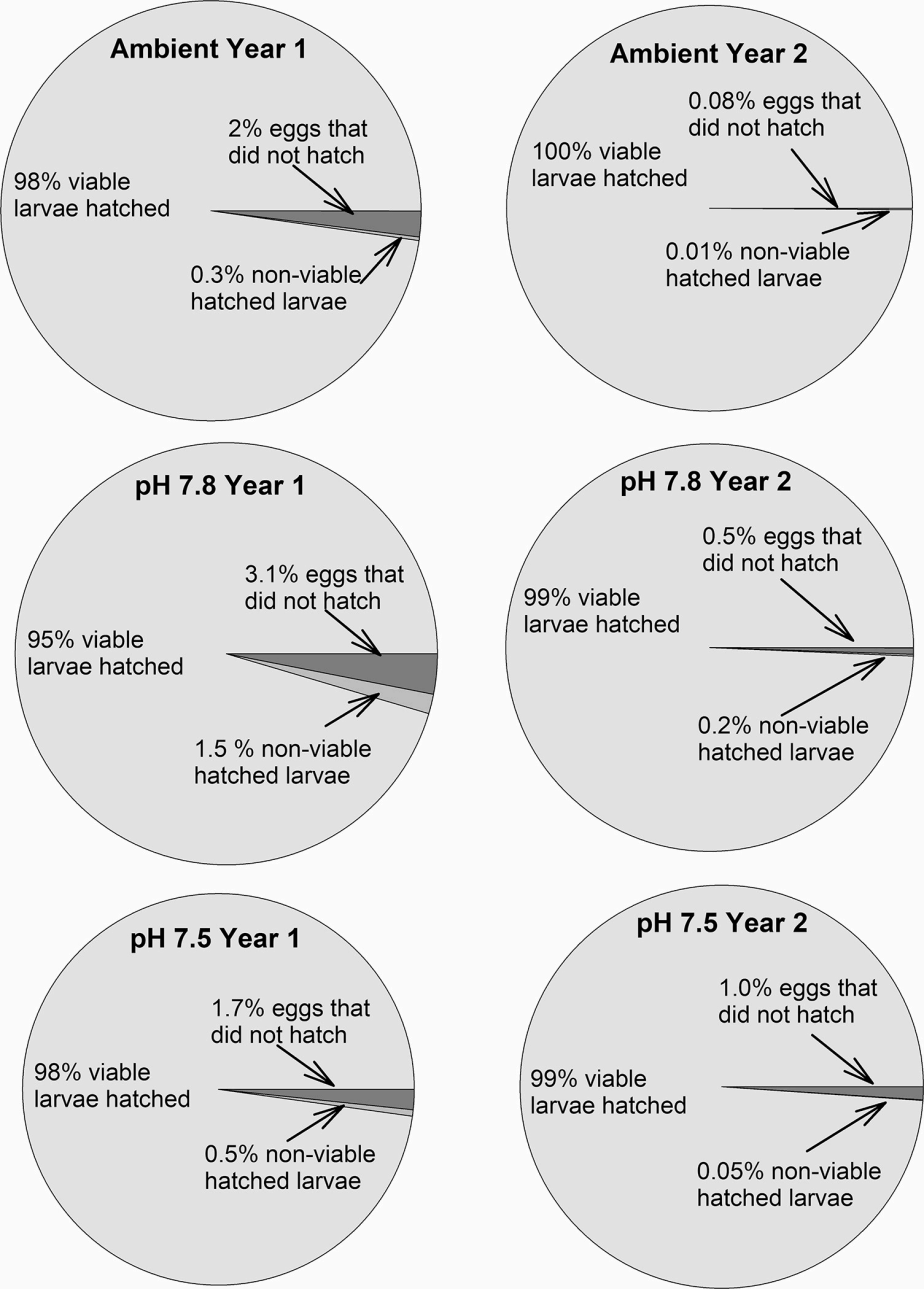
Effect of pH on snow crab hatching success. Hatching success in female snow crabs held in three different pH treatments for 2 years. Values represent the mean in each category. There are no statistically significant differences among treatments in either year.

**Fig 6:**
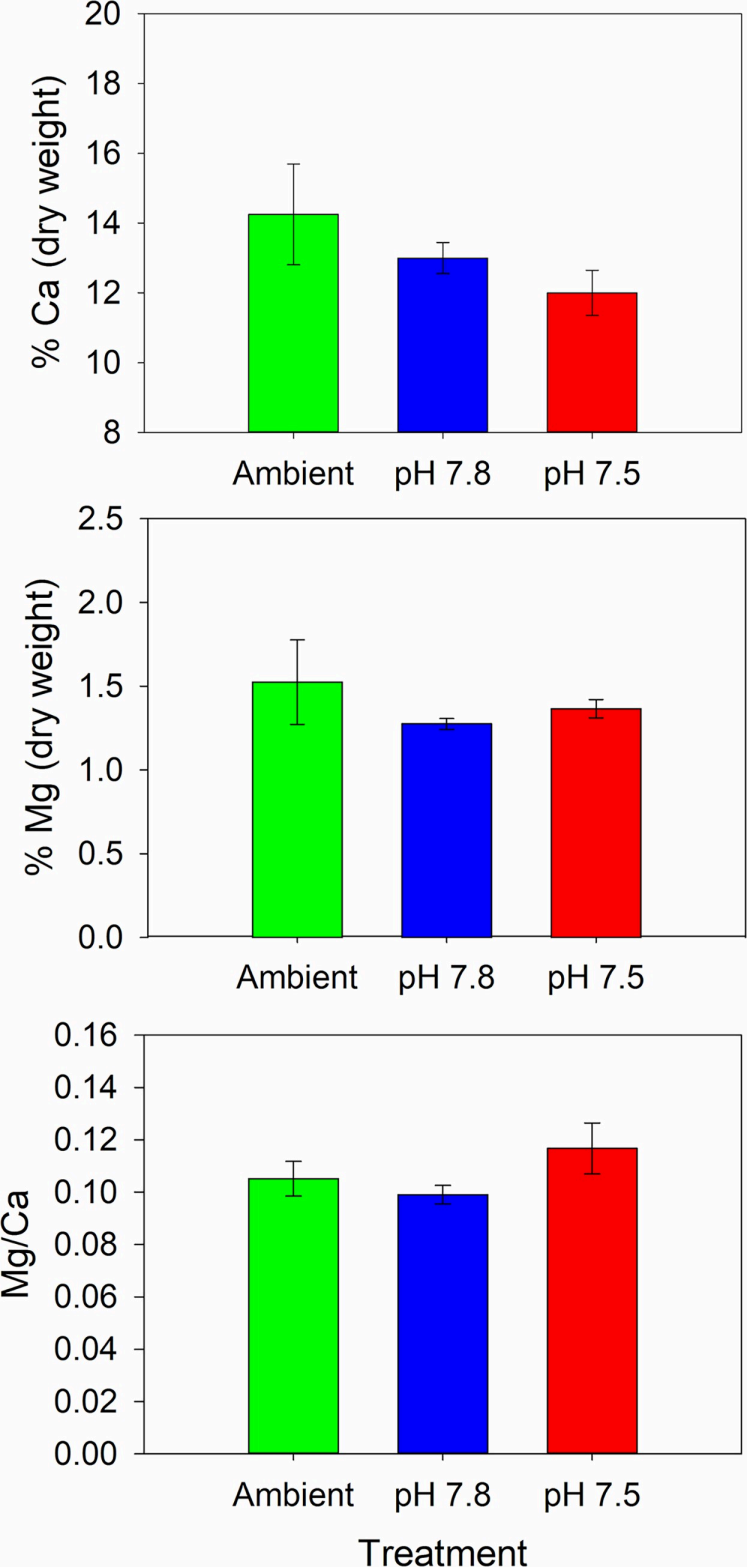
Effect of pH on snow crab carapace mineral content. Calcium and magnesium content and Mg:Ca ratio in the carapaces of female snow crabs held for 2 years in 3 different pH treatments. Error bars are 1 standard deviation. There are no statistically significant differences among treatments.

Fecundity was higher in the second year but did not differ among treatments in either the first or second year (Fig 4, Table 2). Hatching success was high during both years and did not differ among treatments; in year 1 hatching success was greater than 95% in all treatments and in year 2 it was greater than 98% (Fig 5, Table 3). Similarly, the percent of non-viable larvae never averaged above 2% for any treatment and the percent of unhatched eggs was never above 4% (Fig 5, Table 3). The percent Ca and Mg and the Ca:Mg in the female carapaces did not differ among treatments at the end of the experiment (Fig 6, Table 3).

**Table 2:** ANOVA result for effects of water pH on snow crab embryos and females.

	Year 1		Year 2	
	F	p	F	p
**Fecundity**	0.363	0.699	1.415	0.265
**Hatching success**	1.029	0.37^a^	1.656	0.215^a^
**% Non-viable larvae**	2.514	0.099^a^	0.446	0.646^a^
**% Unhatched eggs**	0.508	0.607^a^	1.654	0.215^a^
**% Ca carapace**	—	—	1.896	0.176
**% Mg carapace**	—	—	1.848	0.183^b^
**Ca:Mg carapace**	—	—	2.189	0.138

Summary one-way ANOVA statistics on the effect of low pH on snow crab fecundity, hatching success, and female carapace mineral content. In all cases pH treatment is the factor. Significant deviations from the assumptions of normality are indicated with an ‘a’ and deviations from the assumption of homogeneity of variance are indicated with a ‘b’.

**Table 3:** Water physical and chemical parameters in two years of larval experiments.

	Temperature	pH_F_	pCO_2_	HCO_3_^-^	CO_3_^-2^	DIC	Alkalinity	Ω_Aragonite_	Ω_Calcite_
Treatment	°C		μatm	mmol/kg	mmol/kg	mmol/kg	mmol/kg		
**2015**									
Ambient	3.40 ± 0.17	8.16 ± 0.04	314.79 ± 31.00	1.86 ± 0.02	0.10 ± 0.01	1.98 ± 0.01	2.12 ± 0.01	1.56 ± 0.13	2.49 ± 0.21
pH 7.8	3.17 ± 0.15	7.80 ± 0.02	751.58 ± 24.49	1.99 ± 0.01	0.05 ± 0.00	2.08 ± 0.01	2.12 ± 0.01	0.74 ± 0.02	1.18 ± 0.03
pH 7.5	3.36 ± 0.21	7.50 ± 0.02	1526.01 ± 54.95	2.05 ± 0.01	0.03 ± 0.00	2.16 ± 0.01	2.12 ± 0.00	0.39 ± 0.02	0.62 ± 0.03
**2016**									
Ambient	2.77 ± 0.16	8.14 ± 0.03	322.23 ± 32.83	1.86 ± 0.02	0.10 ± 0.01	1.98 ± 0.01	2.12 ± 0.01	1.48 ± 0.12	2.37 ± 0.19
pH 7.8	2.59 ± 0.17	7.80 ± 0.02	768.68 ± 24.26	1.99 ± 0.00	0.05 ± 0.00	2.08 ± 0.00	2.13 ± 0.01	0.69 ± 0.02	1.11 ± 0.03
pH 7.5	2.75 ± 0.17	7.50 ± 0.02	1556.47 ± 59.39	2.02 ± 0.02	0.02 ± 0.00	2.14 ± 0.02	2.12 ± 0.01	0.36 ± 0.02	0.58 ± 0.02

Water parameters in experimental inserts during larval snow crab experiments. Temperature and pH were measured daily (N = 40 per treatment in 2015 and 52 per treatment in 2016), dissolved inorganic carbon (DIC) and alkalinity were measured weekly (N = 6 per treatment in 2015 and 7 per treatment in 2016), and all other parameters were calculated. Values are mean ± 1 SD.

### Larval experiments

**Fig 7:**
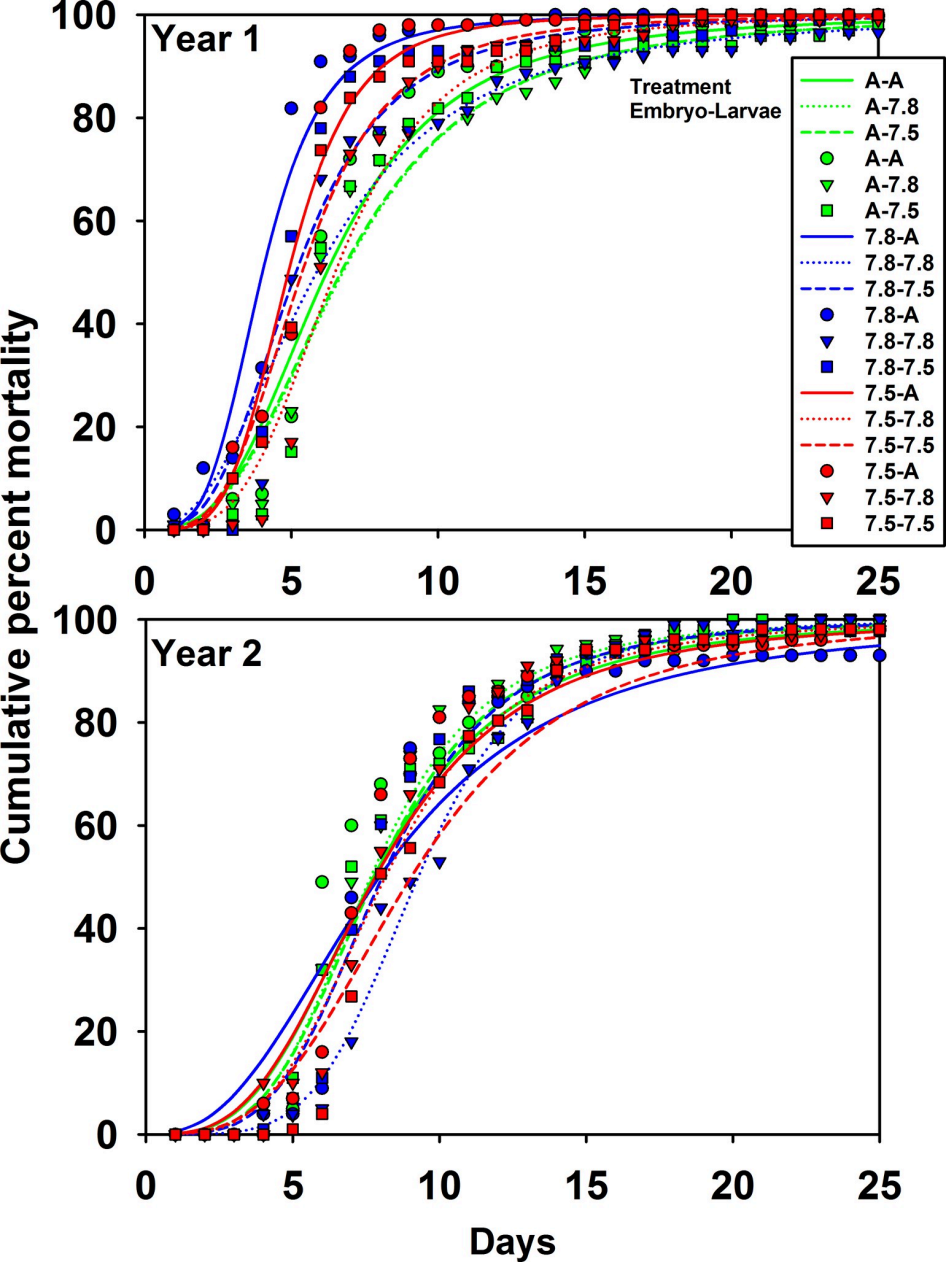
Effect of pH on larval snow crab starvation-survival. Cumulative percent mortality of snow crab larvae in starvation survival experiments in two years of experiments. Treatments represent the pH treatments during the embryo and larval portions of the experiments: A-Ambient pH, 7.8-pH 7.8, 7.5-pH 7.5. Points represent the average of five replicate trials and lines show the best fit curves (see Table 2 for parameter estimates).

In both years the best fit model of starvation survival was one where both the *t*_*50*_ and *s* parameters varied with both embryo and larval pH treatments and their interaction (Table 4). In the first year, embryo treatment had a larger effect than larval treatment with larvae that were in Ambient water as embryos surviving longer than those that were in pH 7.8 or 7.5 water; however, larvae that were held in acidified water as embryos survived longer when exposed to acidified water as larvae than when held in Ambient water (Fig 7, Table 5). In the second year, embryo treatment had almost no effect on larvae that were subsequently held in Ambient water as larvae; however, larvae from embryos held in pH 7.8 and 7.5 water survived longer than their Ambient counterparts when held in acidified water with larvae surviving longest when exposed to the same pH water as larvae as they were when embryos (Fig 7, Table 5).

**Table 4:** Ranking of snow crab larvae starvation survival models.

		Year 1				Year 2			
Model	K	AIC_c_	ΔAIC_c_	Likelihood	AIC_c_ Weight	AIC_c_	ΔAIC_c_	Likelihood	AIC_c_ Weight
*t*_*50*_, *s*	2	3523.66	476.79	0.00	0.00	3943.76	235.03	0.00	0.00
*t*_*50*_*(E)*, *s*	4	3344.38	297.51	0.00	0.00	3899.79	191.06	0.00	0.00
*t*_*50*_*(L)*, *s*	4	3381.45	334.58	0.00	0.00	3947.29	238.56	0.00	0.00
*t*_*50*_*(E*,*L)*, *s*	6	3158.61	111.74	0.00	0.00	3903.04	194.31	0.00	0.00
*t*_*50*_*(E#L)*, *s*	10	3120.76	73.89	0.00	0.00	3850.76	142.03	0.00	0.00
*t*_*50*_, *s(E)*	4	3448.34	401.47	0.00	0.00	3922.66	213.92	0.00	0.00
*t*_*50*_, *s(L)*	4	3502.29	455.41	0.00	0.00	3861.80	153.07	0.00	0.00
*t*_*50*_, *s(E*,*L)*	6	3416.76	369.89	0.00	0.00	3847.21	138.48	0.00	0.00
*t*_*50*_, *s(E#L)*	10	3401.75	354.88	0.00	0.00	3803.82	95.09	0.00	0.00
***t***_***50***_ ***(E#L)*, *s(E#L)***	**18**	**3046.87**	**0.00**	**1.00**	**1.00**	**3708.73**	**0.00**	**1.00**	**1.00**

Models of snow crab larvae starvation survival ranked by Akieke’s Information Criterion with the best-fit model indicated by bold font. Model indicates the parameters in each model; parameters were linear function of factors where they are included parenthetically (see text for model details). Factors included pH treatment during embryo development (E) and during the larval experiments (L). K indicates the number of parameters in each model.

**Table 5:** Snow crab larval starvation survival model parameters.

**Embryo**	A	A	A	7.8	7.8	7.8	7.5	7.5	7.5
**Larvae**	A	7.8	7.5	A	7.8	7.5	A	7.8	7.5
	**Year 1**								
***t***_***50***_	6.20	6.75	6.69	4.02	5.86	5.15	4.90	6.49	5.36
***s***	-3.06	-2.92	-2.89	-2.92	-2.48	-3.21	-4.38	-3.69	-3.61
	**Year 2**								
***t***_***50***_	7.75	7.65	7.81	7.95	9.27	8.05	7.82	8.15	9.03
***s***	-3.32	-3.92	-3.78	-3.92	-4.85	-3.98	-3.21	-3.74	-3.27

Parameter estimates for the best-fit model of snow crab larvae survival in starvation-survival experiments. Embryo and Larvae indicate the pH treatment at each stage. Parameters include the time to 50% mortality (*t*_*50*_) in days and a unitless slope parameter (*s*). See text for model details.

**Fig 8:**
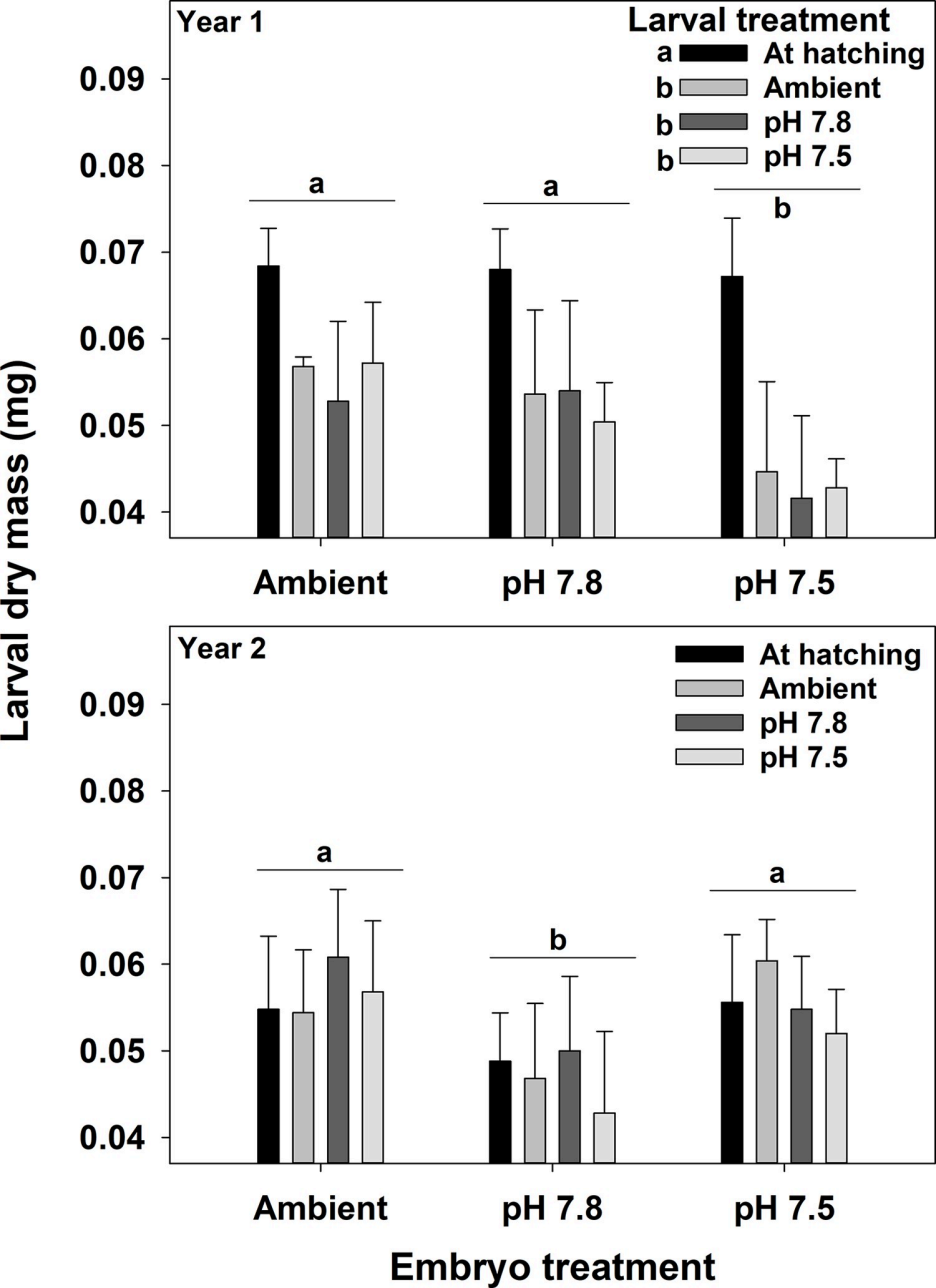
Water pH and snow crab larval mass. Effects of holding pH during the embryo and larval stages on the dry mass of snow crab larvae. Bars are means + 1 standard deviation. For larval treatments, “At hatching” represents larvae sampled immediately after hatching and Ambient, pH 7.8, and pH 7.5 represent larvae held at those pH treatments for 7 days. Statistically significant differences (Tukey’s test) among treatments are indicated with different letters.

**Fig 9:**
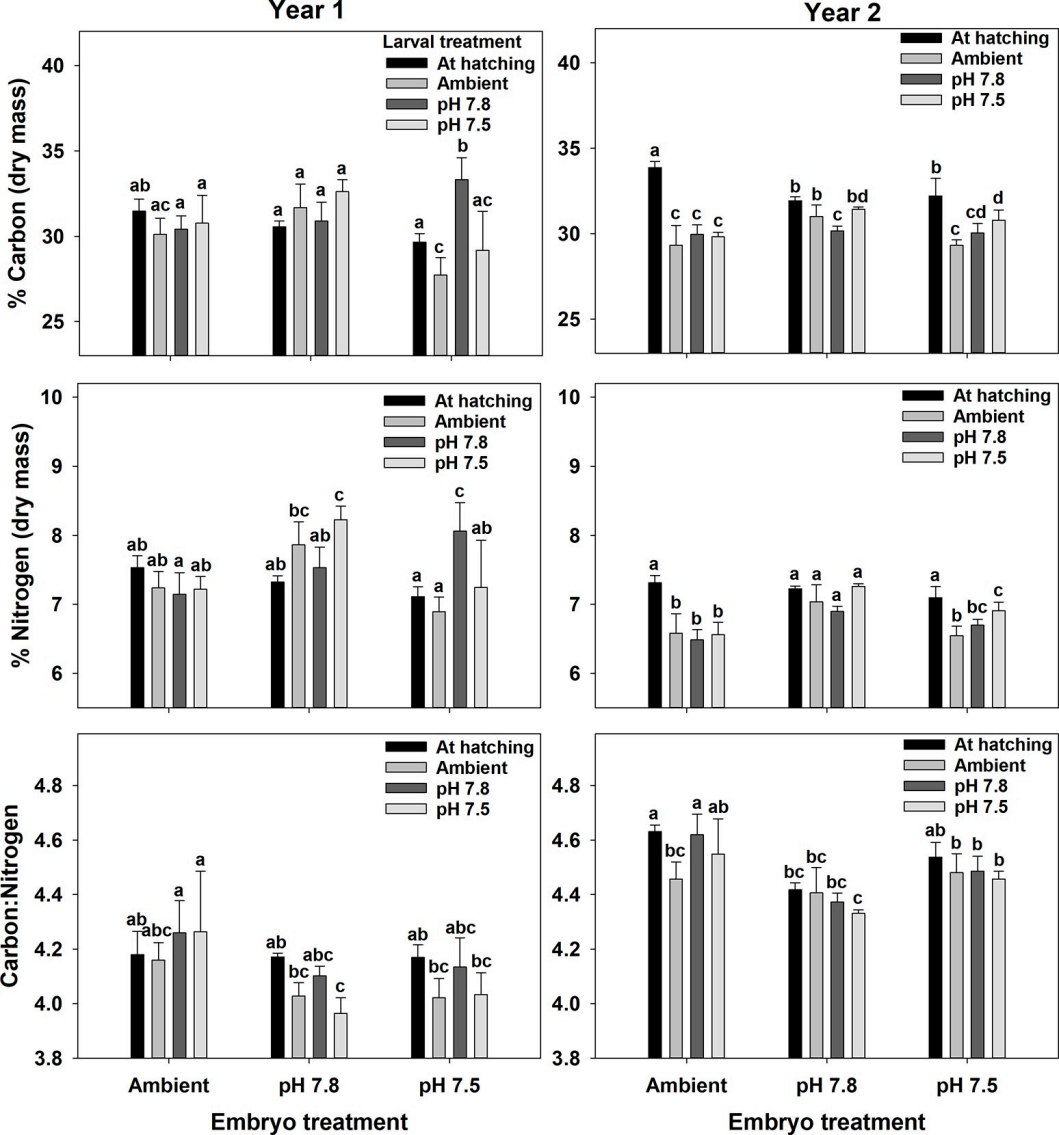
Water pH and snow crab larval elemental composition. Effects of holding pH during the embryo and larval stages on the carbon and nitrogen contents and the C:N ratio of snow crab larvae. Bars are means + 1 standard deviation. For larval treatments, “At hatching” represents larvae sampled immediately after hatching and Ambient, pH 7.8, and pH 7.5 represent larvae held at those pH treatments for 7 days. Statistically significant differences (Tukey’s test) among treatments are indicated with different letters.

**Fig 10:**
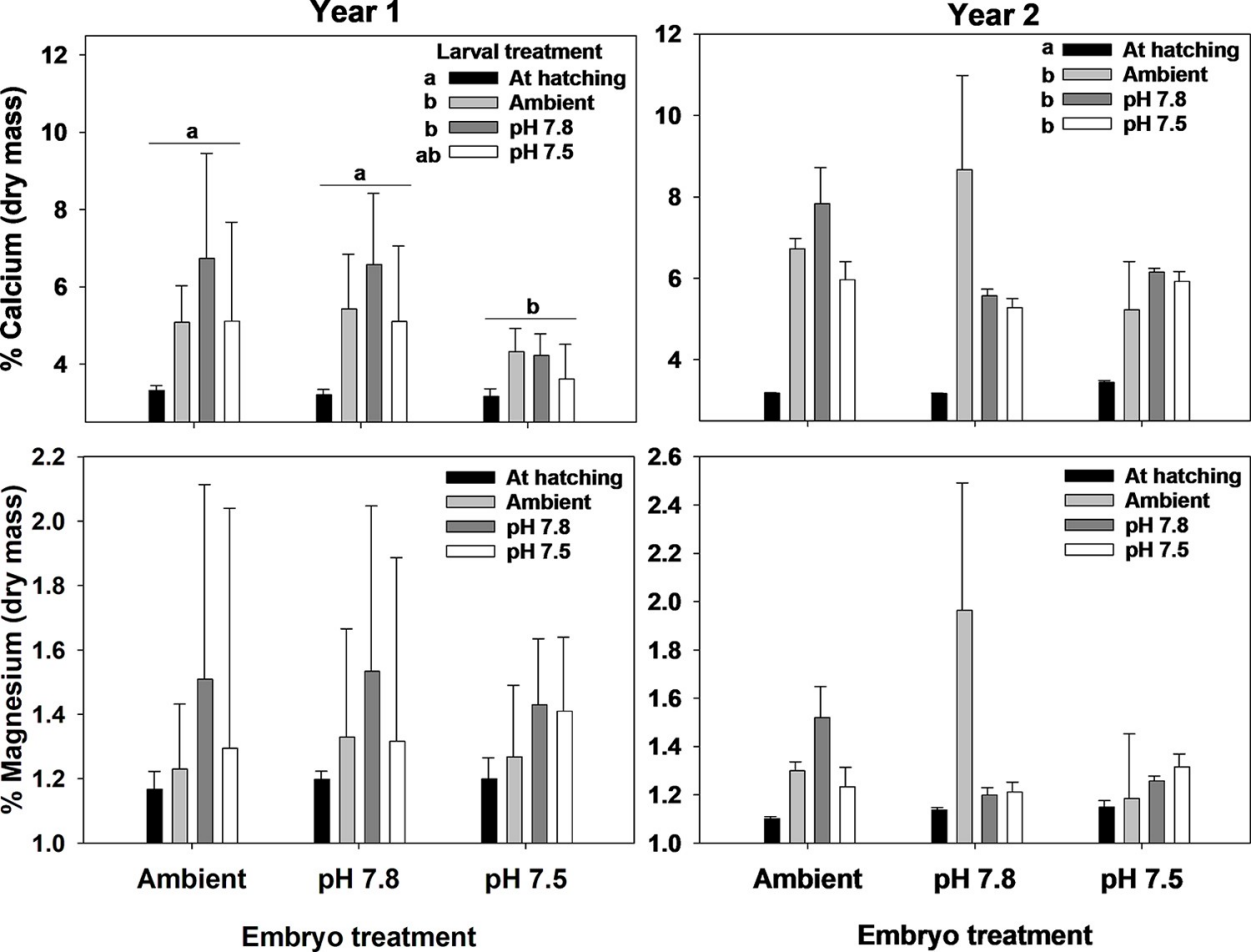
Water pH and snow crab larval calcium and magnesium content. Effects of holding pH during the embryo and larval stages on the calcium and magnesium contents of snow crab larvae. Bars are means + 1 standard deviation. For larval treatments, “At hatching” represents larvae sampled immediately after hatching and Ambient, pH 7.8, and pH 7.5 represent larvae held at those pH treatments for 7 days. Statistically significant differences (Tukey’s test) among treatments are indicated with different letters.

In the first year, larval dry mass varied with both embryo and larval treatment and in the second year it varied only among embryo treatment (Fig 8, Table 6). In the first year, larval mass was higher at hatching than after 7 days of holding but there was no difference among the three larval pH treatment. Additionally, larvae that hatched from embryos held at pH 7.5 had lower dry mass than those in the other two treatments and were about 19% lower than the Ambient treatment. In the second year, larvae that hatched from embryos held at pH 7.8 had lower dry mass than the other two treatments and were about 17% lower than the Ambient treatment. Carbon and nitrogen content and the C:N ratio varied among both embryo and larval treatments and their interactions in both years (Fig 9, Table 6). Although the differences were statistically significant, there were no clear trends in the data either within or among years that showed consistent effects of either embryo or larval treatment and effect sizes were generally small (>5% differences). Calcium content varied among embryo and larval treatments in the first year but only among larval treatments in the second year (Fig 10, Table 6). In the first year, there was a significant increase in calcium content between the larvae at hatching and after 7 days of holding with a trend for larvae held at pH 7.5 showing a non-significant increase. Further, larvae hatched from embryos held at pH 7.5 had lower calcium content than those from the other two treatments. In the second year, the only difference was a significant increase in calcium content between larvae at hatching and those held for 7 days; there were no differences among embryo or larval pH treatments. There were no significant differences in magnesium content among either embryo or larval treatments.

**Table 6:** Summary ANOVA results for snow crab larval experiments.

	Year 1						Year 2					
	Embryo		Larval		E*L		Embryo		Larval		E*L	
	F	p	F	p	F	p	F	p	F	p	F	p
**C**	7.547	0.001	5.233	0.003	8.829	< 0.0005	3.804	0.029	61.894	< 0.0005	9.948	< 0.0005
**N**	12.854	0.000	3.030	0.038	9.309	< 0.0005	31.240	0.000	36.009	< 0.0005	6.929	< 0.0005
**C:N**	13.729^a^	0.000	5.497	0.003	2.605	0.029	37.567	0.000	5.417	0.003	2.386	0.043
**Ca**	4.500^a^	0.016	7.619	< 0.0005	0.735	0.624	0.855^a^	0.432	12.031	< 0.0005	2.000	0.084
**Mg**	0.028^a^	0.973	1.148	0.340	0.115	0.994	0.730^a,b^	0.487	2.061	0.118	1.962	0.090
**Mass**	9.291	< 0.0005	22.689	< 0.0005	1.145	0.352	9.995	< 0.0005	1.038	0.384	0.860	0.531

Results of ANOVA for the effects of exposure to low pH at the embryo (E) and larval (L) stages on snow crab embryo in the first and second year of the experiments. Separate analyses were done for each year. Responses include carbon (C), nitrogen (N), calcium (Ca), and magnesium (Mg) contents; the C to N ratio; and the average larval dry mass (Mass). Significant deviations from the assumptions of normality are indicated with an ‘a’ on the first F statistic for each ANOVA and deviations from the assumption of homogeneity of variance are indicated with a ‘b’.

**Fig 11:**
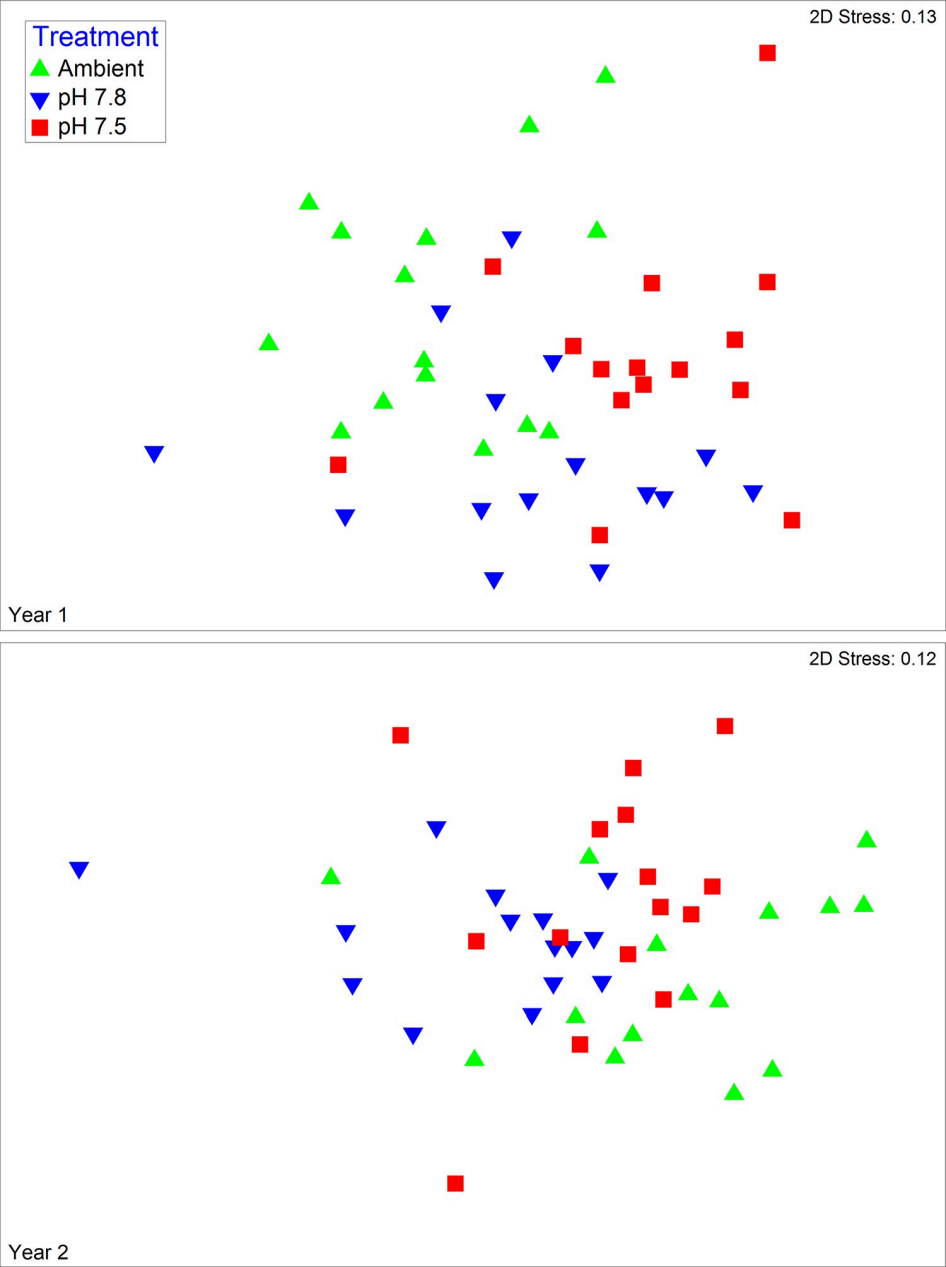
Effect of water pH on snow crab larvae morphometrics. Non-metric multidimensional scaling plots of morphometrics of snow crab larvae hatched from larvae held in three different pH waters in the first and second years of the experiment.

Larval morphometry differed among treatments in both the first (Global R = 0.232, p < 0.0005) and second (Global R = 0.223, p < 0.0005) years (Fig 11). Despite the statistical significance the low Global R value each year suggested actual differences among treatments were low. SIMPER analysis showed that the rostral-dorsal length and carapace width were the most important contributors to differences between treatment pairs in both years contributing to >50% of the difference between treatments in all cases (Table 7). In both years, larvae from embryos reared in Ambient water were in general slightly larger than those reared in lower pH waters in these metrics. However, effect sizes were small and smaller in the second than in the first year; effect sizes averaged 4.6% in the first year with a maximum of 13% whereas they averaged 3.4% in the second year with a maximum of 7.3%.

**Table 7:** SIMPER analysis results for effect of water pH on snow crab larval morphometry.

Year 1					Year 2				
**Ambient vs. pH 7.8**									
	**Mean (mm)**					**Mean (mm)**			
**Variable**	**Ambient**	**pH 7.8**	**% Contribution**	**% Difference**	**Variable**	**Ambient**	**pH 7.8**	**% Contribution**	**% Difference**
CW	2.89	2.73	38.7	5.5	RDL	5.15	4.86	52.1	5.6
PL	1.68	1.83	20.3	-8.9	DSL	2.18	2.07	14.9	5.0
RDL	5.24	5.2	14.0	0.8	CW	2.74	2.63	12.5	4.0
LSL	0.965	0.884	12.4	8.4	RSL	1.79	1.66	10.9	7.3
DSL	2.25	2.2	8.1	2.2	PL	1.78	1.71	6.2	3.9
RSL	1.67	1.74	6.6	-4.2	LSL	0.91	0.905	3.5	0.5
**Ambient vs. pH 7.5**									
	**Mean (mm)**					**Mean (mm)**			
**Variable**	**Ambient**	**pH 7.5**	**% Contribution**	**% Difference**	**Variable**	**Ambient**	**pH 7.5**	**% Contribution**	**% Difference**
CW	2.89	2.68	34.5	7.3	RDL	5.15	5	35.9	2.9
RDL	5.24	5.07	24.5	3.2	CW	2.74	2.8	16.3	-2.2
LSL	0.965	0.84	14.7	13.0	DSL	2.18	2.12	16.0	2.8
PL	1.68	1.76	11.2	-4.8	RSL	1.79	1.66	14.9	7.3
DSL	2.25	2.14	9.3	4.9	LSL	0.91	0.923	9.7	-1.4
RSL	1.67	1.67	5.8	0.0	PL	1.78	1.78	7.3	0.0
**pH 7.8 vs. pH 7.5**									
	**Mean (mm)**					**Mean (mm)**			
**Variable**	**pH 7.8**	**pH 7.5**	**% Contribution**	**% Difference**	**Variable**	**pH 7.8**	**pH 7.5**	**% Contribution**	**% Difference**
CW	2.73	2.68	34.8	1.8	RDL	4.86	5	37.9	-2.9
RDL	5.2	5.07	22.6	2.5	CW	2.63	2.8	26.0	-6.5
LSL	0.884	0.84	13.8	5.0	PL	1.71	1.78	11.3	-4.1
PL	1.83	1.76	10.5	3.8	RSL	1.66	1.66	9.8	0.0
RSL	1.74	1.67	9.3	4.0	DSL	2.07	2.12	8.4	-2.4
DSL	2.2	2.14	9.0	2.7	LSL	0.905	0.923	6.6	-2.0

Similarity percentage analysis of snow crab larvae morphometric measurements from larvae hatched from embryos held in water at three different pHs. Variables include carapace width (CW), lateral spine length (LSL), dorsal spine length (DSL), rostro-dorsal length (RDL), rostral spine length (RSL), and protopodite length (PL). Mean measurements for each treatment within each year are calculated. Variables are arranged by their percent contribution to differences between treatment comparisons. The % Difference represents the average percent difference between the two treatments with negative numbers indicating the second treatment is larger.

## Discussion

In this study, snow crabs were held for two years over two brooding cycles at low pH with no detectible effects on embryo development, larval hatching, or female calcification. This demonstrates that both the direct effects of low pH and the maternal effects (i.e. carryover effects from oogenesis) on embryos are negligible. Further, a companion paper found no difference among treatments in a detailed analysis of the microstructure and function of the cuticle in the carapace and chela of these females [69]. Combined, this suggests that the mature females are physiologically well adapted to low pH, at least when fed *ad libitum*. Additionally, snow crab larvae were also tolerant of low pH conditions suggesting that this life-history stage of this species is well adapted to a broad range of pH conditions. In the larval experiments, we examined starvation survival and larval condition and were able to parse out direct effects from the carryover effects from oogenesis and embryogenesis. In general, for larvae, effect sizes were small or non-significant and positive carryover effects from earlier life history stages reduced or eliminated the negative effects of OA suggesting the species is able to acclimate to large carbonate chemistry perturbations. We concluded that snow crab adults, embryos, and larvae may be highly resistant in the face of changing oceanic carbonate chemistry, although other life-history stages not examined may be vulnerable and interactive effects with other stressors, particularly increased temperature are possible.

There were no detectible direct effects of low pH on embryos in this experiment; rate of development, embryo morphometrics, and survival/hatching success were similar across all treatments. Direct effects of OA on embryos are relatively rare in decapods; a fair number of species show no direct negative effects on measured parameters including the fiddler crab *Leptuca thayeri* [70], the Norway lobster *Nephrops norvegicus* [71, 72], and the spider crab *Hyas araneus* [35]. In other species low pH has minor effects on parameters, such as hatch timing or duration, which are unlikely to have a strong effect on overall offspring fitness, without affecting embryo development or survival [13, 73]. To our knowledge, in only one species have substantial direct negative effects of OA on embryo development and hatching success been detected: the stone crab, *Menippe mercenaria* [12]. Most decapods carry embryos packed tightly together on pleopods and must regularly aerate the clutch to prevent it from becoming hypoxic; however, in between aerations the dissolved oxygen drops in the egg mass [74] and, presumably, the CO_2_ increases commensurately. In blue king crab, *Paralithodes platypus*, O_2_ level varies with location in the egg mass with eggs in the center experiencing O_2_ saturations as low as 50% [74]. Thus the embryo stages of crabs likely need to be well adapted to substantial variance in *pCO*_2_ levels and this may explain why this life history stage appears to be highly tolerant of OA conditions across many taxa.

Not only were no direct effects observed, but there were no negative maternal effects of high *pCO*_2_ on snow crab embryos. Carryover effects, when exposure to a stressor at one life history stage affects fitness at a subsequent stage, from OA are relatively common. For example, in Tanner crabs, exposure to high *pCO*_2_ during oogenesis had substantial negative carryover effect on both embryos [13] and the subsequent larvae [53]. High *pCO*_2_ causes changes to gene expression and disrupts oocyte development in Chinese mitten crabs, *Eriocheir sinensis* suggesting direct effects on oogenesis could be a mechanism for these carryover effects [75]. Alternatively, high *pCO* could simply increase the energy requirement to maintain homeostasis and thus decrease the energy available for other processes [76] including reproduction. Regardless, the lack of an effect in snow crabs, both in terms of oogenesis and in terms of shell maintenance (i.e. Ca and Mg content) or cuticle properties [69], show that this species has the physiological and energetic plasticity to maintain somatic and reproductive processes, at least all that have been measured thus far, under hypercapnic stress. Positive carryover effects from OA exposure during both oogenesis and embryogenesis in snow crab larvae suggests that physiological plasticity is a major contributor.

There were no negative direct effects of exposure to low pH on snow crab larvae on any of the metrics measured here. This can be inferred by examining the differences (or lack thereof) among larval treatments within the embryos reared at Ambient pH. Exposure to low pH as larvae did not affect mass, carbon or nitrogen content, or calcium or magnesium content. Further, the effect on starvation survival time was slightly positive, increasing it by about half a day or around 10% in the first year, although not the second. Starvation survival time is a function of energy storage and metabolic rate such that a decrease suggests that either the larvae had less energy storage to start with or the metabolic rate was increased, or some combination. Because all the larvae came from the same pool they would have started with the same amount of energy storage on average so any differences among larval treatments (direct effects) were mostly likely caused by differences in metabolic rate. This is consistent with the response of Tanner crab larvae in virtually identical experiments [53]. Effects of ocean acidification on crustacean larvae are mixed, there are many species whose larval phases are tolerant of low pH [15, 77, 78], but there are examples to the contrary [16, 36, 79]. It is not clear what factors are involved in determining the level of sensitivity to low pH; however, there is evidence that some species lack plasticity at the larval phase. Red king crab larvae, for example, have decreased starvation survival and carbon content when held at low pH [36], but they do not alter their gene expression patterns [80]. Molecular physiology in snow crab larvae should be investigated to determine if they alter their physiology and, if so, what pathways they use.

Carryover effects from exposure to low pH at the embryo stage were generally larger than the direct effects. Such exposure reduced larval mass and size and substantially reduced starvation survival time by over a third in the first year. These results are also consistent with those of Tanner crabs where carryover effects from exposure at the embryo stage were larger than direct effects although effects sizes on snow crab were smaller [53]. Negative carryover effects, where exposure at one life history stage reduces performance at another stage is a common response [35, 81, 82]. In these cases, it is likely that increased energetic demands at these earlier stages, although they may not directly affect the earlier stage, affect processes later on.

However, snow crab larvae also experienced positive carryover effects. In the first year, the negative carryover effect on starvation survival was reduced or eliminated when the larvae were also held at low pH. This suggests that plastic processes at the embryo stage altered the larval physiology in ways that increased fitness in low pH. Further, positive maternal effects (based on a comparison of the results of the second year with those of the first) either eliminated or reduced, to the point where they were likely biologically insignificant, the negative carryover effects from embryo development on most of the parameters we measured including starvation survival, morphometry, and calcium content. And, beyond that, the carryover effects from oogenesis were additive to the physiological acclimation that occurred during embryo development such that, in the second year, larvae that were hatched from females that had been held at low pH throughout egg and embryo development survived substantially longer than those held at Ambient throughout. In general, in both years, the larvae survived longest when the embryo treatments and larval treatments were the same and the effect was stronger in the second year. The pH in snow crab habitat varies substantially both seasonally and spatially [55, 56] and this result suggests that under reduced pH conditions maternal and embryonic exposure to low pH primes larvae to do better in similar conditions. We explore potential mechanisms of this in the next paragraph; however, this plastic response, operating both through maternal and through carryover effects from embryogenesis, suggests that this may be an adaptive response as long as pH in the maternal/embryo habitat correlates with that of the larvae. However, if there is a mismatch, this could end up being maladaptive [e.g., 83] if there are tradeoffs between tolerance to stressful conditions and other metrics of larval health. In the second year, the starvation survival of larvae hatched from the pH 7.5 treatment was longer than the larvae from the ambient treatment under all conditions; this strongly suggests that there are likely tradeoffs, such as a lower metabolic rate, lower activity levels, or slower growth, otherwise the larvae hatched from the ambient treatment would poorer adapted to ambient water than those from pH 7.5, which seems unlikely. Given that we have little data, particularly across years, it is impossible to address whether there is a correlation between the embryo and larval pHs in the field at this point.

It’s not clear what mechanism is involved in conferring the positive maternal effects. It could be selection, through selective mortality of females poorly adapted to low pH, or transgenerational plasticity [37]. Transgenerational and selective breeding experiments suggest that many species have the capacity to evolutionarily adapt to ocean acidification via natural selection [84–88], although other mechanisms may also contribute. In the case of snow crabs, the mortality over the course of the experiment did not vary much between the treatments which makes this a less likely, though still possible, mechanism. Alternatively, a stress response in the females could induce greater maternal investment in oocytes. The size and lipid content of crab eggs is a plastic response that can vary within and among populations and in response to environmental variables [89, 90]; increased maternal investment can increase larval survival [91]. Other taxa, including oysters, increase the energetic content of eggs in response to OA [92]. This is not well supported in the case of snow crabs as larval size (both in terms of mass and length) did not differ between embryos reared in Ambient and pH 7.5 water in the second year and the carbon content was actually slightly lower in pH 7.5. Another potential mechanism is increased antioxidant activity [37]. OA leads to oxidative stress and, in some species increased antioxidant activities [93, 94], which can have positive transgenerational effects. For example, in the copepod *Acartia bifilosa*, mature females increased their oxidative stress response in response to OA and eggs of females with the highest oxidative stress response had higher hatching success rates [95]. As we did not measure oxidative stress or indicators of stress response this could be mechanism in this case but more research is needed. A likely response is a decreased metabolic rate which many species show as an adaptive response that decreases extracellular pCO_2_ [96] and can increase survival [97, 98]. The increased starvation-survival of the low pH treatment larvae in our experiment suggests that this is a likely mechanism given that there were no differences in larval mass or carbon content. Finally, epigenetic changes, particularly changes in DNA methylation can be induced by exposure to ocean acidification [99, 100] and can be an adaptive response [101] and this possibility should be investigated further in snow crabs. Since these responses are not mutually exclusive any or all of them could be factors in this case.

The difference in the response to high *pCO*_2_ between Tanner and snow crabs is striking, particularly in regards to the response of the embryos. These two species are congenators, have overlapping distributions in Bering Sea [102], and are able to produce fertile hybrids [103, 104]. However, OA reduces hatching success in Tanner crab by more than 70%, reduces calcium content in larvae, increases mortality of both adults and juveniles, reduces juvenile growth, increases hemocyte mortality, and causes both internal and external dissolution of the adult carapace [13, 22, 24, 52–54]. Although fewer studies have been done on snow crab, in the comparable studies that have been done (including this one) no significant negative effects have been observed. Although the difference between these two species is large, this is not unprecedented; different populations of the same crab species, *Hyas araneus*, show a different response to high *pCO*_2_ [25, 105]. However, the strong difference in the responses and the similarity of the species opens up the opportunity to explore how their physiological differences contribute to their sensitivity/tolerance of high *pCO*_2_. Future work should focus on examining how high *pCO*_2_ affects blood chemistry, respiration rate, and gene expression in both species.

Given the number of strongly negative effects we’ve documented over the years, it is, in all honesty, a nice change for us to be able to report relatively good news in regards to how high *pCO*_2_ will affect a commercial crab species. With the data currently available we are cautiously optimistic that snow crab are likely to prove resistant in the face of changing oceanic carbonate chemistry. Future work, however, should still be conducted as different life-history stages may respond very differently to OA; larval American lobsters (*Homarus americanus*) are highly resistant whereas juveniles are very sensitive [106] and European lobster (*Homarus gammarus*) have both sensitive and resistant larval stages and a very sensitive juvenile stage [107, 108]. Thus, studies examining all larval stages as well the juvenile stage of snow crab should be performed before it can be concluded that the species as a whole will be resistant to OA. Finally, as *pCO*_2_ in the oceans increases, temperatures are also projected to increase so experiments examining the separate and interactive effects of *pCO*_2_, temperature, and hypoxia on snow crab are also called for as these can alter the effects of OA on species [105, 109, 110].
